# Supplementary material for: Patterns of mortality in domesticated ruminants in Ethiopia
Source: Front Vet Sci. 2022 Nov 24;9:986739. doi: 10.3389/fvets.2022.986739 (PMC9729939; doi:10.3389/fvets.2022.986739)
Supplement: Supplementary file 1 [file Data_Sheet_1.PDF]

frontiers

Supplementary Material

Farmer Survey for cattle owners

Surveillance of major infectious and reproductive diseases of cattle

DEMOGRAPHIC DATA

1. Name (optional) ………………………………………… phone number …………………………………

2. (Location) State…………………… Local Government Area…………………………………………

3. Age ……………………………… Educational level …………………………………………………………

4. How long have you been keeping ruminants? ……………………………………………………………

5. What kind of animals do you keep (Please Tick as appropriate) Beef Cattle ☐ Dairy Cattle ☐

MORTALITY AND REPRODUCTIVE LOSSES

Serial	Questions	Bulls	Lactating cow	Dry cows	Heifers	Steers	Calves	Oxon
6	Average herd composition last year							

Serial	Questions	Bulls	Lactating cow	Dry cows	Heifers	Steers	Calves	Oxon
7	Animals dead last year							
	Tryps							
	FMD							
	LSD							

	**Infectious respiratory diseases:**<br>***CBPP**<br>***Pasteurellosis**<br>***Other infectious respiratory diseases**							
	**Injuries**							
	**Nutrition (insufficient food/water)**							
	**GI parasites**							
	**Other respiratory**							
	**Other digestive**							
	**Other reproductive**							
	**Sudden death**							
	**Regular slaughter**							
	**Other - unspecific**							

Serial	Questions	Bulls	Lactating cow	Dry cows	Heifers	Steers	Calves	Oxon
8	Number of abortions last year							

	**Clinical signs compatible with Brucellosis**							
	**Clinical signs compatible with FMD**							
	**Clinical signs compatible to other diseases**							
	**Linked to heat stress**							
	**Linked to injuries and other causes**							
	**Linked to poor nutrition**							
	**Early abortions (< 5 months)**							
	**Late abortions (> 5 months)**							

**Serial**	**Questions**	**Bulls**	**Lactating cow**	**Dry cows**	**Heifers**	**Steers**	**Calves**	**Oxon**
**9**	**Animals sold because illness last year**							
	**Tryps**							
	**LSD**							
	**FMD**							

	**Infectious respiratory diseases:**<br><br>***CBPP**<br><br>***Pasteurellosis**<br><br>***Other infectious respiratory diseases**							
	**Injuries**							
	**Nutrition (insufficient food/water)**							
	**GI parasites**							
	**Other respiratory**							
	**Other digestive**							
	**Other reproductive**							
	**Other - unspecific**							

Thank You for Your Participation

Farmer Survey for sheep and goat owners

Surveillance of major infectious and reproductive diseases of small ruminants

DEMOGRAPHIC DATA

1. Name (optional) ……………………………………… phone number ………………………………

2. (Location) State…………………… Local Government Area…………………………………………

3. Age ……………………………… Educational level ………………………………………………………

4. How long have you been keeping ruminants? ……………………………………………………………

5.1 What kind of animals do you keep (Please Tick as appropriate) Sheep ☐ Goat ☐

Serial	Question	Sheep	Goat
5.2	Total population last year		

MORTALITY AND REPRODUCTIVE LOSSES

Serial	Question	< 3m male	< 3m female	3m - 1 yr male	3m - 1 yr female	> 1 yr male	> 1 yr female
6	Average herd composition last year						

Serial	Questions	< 3m male	< 3m female	3m - 1 yr male	3m - 1 yr female	> 1 yr male	> 1 yr female
7	Animals dead last year						
	Tryps						
	Orf						
	FMD						

	**Infectious respiratory diseases:**<br><br>***CCPP**						
	***PPR**						
	***Pasteurellosis**						
	***Other infectious respiratory diseases**						
	**Injuries**						
	**Foot rot**						
	**Nutrition (insufficient food/water)**						
	**GI parasites**						
	**Other respiratory**						
	**Other digestive**						
	**Other reproductive**						
	**Sudden death**						
	**Other - unspecific**						

Serial	Questions	< 3m male	< 3m female	3m - 1 yr male	3m - 1 yr female	> 1 yr male	> 1 yr female
8	Number of abortions last year						
	Clinical signs compatible with Brucellosis						
	Clinical signs compatible with FMD						
	Clinical signs compatible with PPR						
	Clinical signs compatible to other diseases						
	Linked to heat stress						
	Linked to injuries and other causes						
	Linked to poor nutrition						

Serial	Questions	< 3m male	< 3m female	3m - 1 yr male	3m - 1 yr female	> 1 yr male	> 1 yr female
9	Animals sold because illness last year						
	Tryps						
	Orf						

	**FMD**						
	**Infectious respiratory diseases:**<br><br>***CCPP**						
	***PPR**						
	***Pasteurellosis**						
	***Other infectious respiratory diseases**						
	**Injuries**						
	**Foot rot**						
	**Nutrition (insufficient food/water)**						
	**GI parasites**						
	**Mange**						
	**Other respiratory**						
	**Other digestive**						
	**Other reproductive**						
	**Other - unspecific**						

Thank You for Your Participation
